# Regional citrate anticoagulation in CVVH: a new protocol combining citrate solution with a phosphate-containing replacement fluid

**DOI:** 10.1186/cc10973

**Published:** 2012-03-20

**Authors:** S Morabito, V Pistolesi, L Tritapepe, E Vitaliano, E Strampelli, F Polistena, L Zeppilli, A Pierucci

**Affiliations:** 1 Policlinico Umberto I, Rome, Italy; 2 Pertini H, Rome, Italy

## Introduction

Regional citrate anticoagulation (RCA) is a highly effective anticoagulation (AC) method in CRRT and different combinations of citrate (Citr) and CRRT solutions can affect the acid-base (A-B) balance. Regardless of the AC protocol, hypophosphatemia occurs frequently in CRRT (80%). The aim was to evaluate safety and effects on A-B balance of a new RCA-CVVH protocol using 18 mmol/l Citr solution combined with a phosphate-containing hemofiltration (HF) solution.

## Methods

In our center, RCA-CVVH is routinely performed with a 12 mmol/l predilution Citr solution (Prismocitrate 10/2) and a postdilution HF solution (HCO_3_^- ^32, Ca^2+ ^1.75, Mg^2+ ^0.5, K^+ ^2 mmol/l) (protocol A). In the case of persistent acidosis, not related to Citr accumulation, NaHCO_3 _infusion is started. In order to optimize the buffer balance, a new protocol has been designed throughout a mathematical model developed to estimate Citr and HCO_3_^- ^mass transfer. Recently introduced solutions have been adopted: 18 mmol/l predilution Citr solution (Prismocitrate 18), postdilution HF solution (Phoxilium, HCO_3_^- ^30, phosphate 1.2, Ca^2+ ^1.25, Mg^2+ ^0.6, K^+ ^4 mmol/l) (protocol B). In relation to Qb, the Citr solution rate was set to meet the target circuit Citr concentration (3 mmol/l). To maintain systemic Ca^2+ ^(1.1 to 1.25 mmol/l), CaCl_2 _10% was started according to estimated Ca^2+ ^loss.

## Results

In a cardiac surgery patient with AKI, A-B status and electrolytes have been evaluated comparing protocol A (five circuits, 301 hours) versus protocol B (two circuits, 97 hours): pH 7.39 ± 0.03 versus 7.44 ± 0.03 (*P *< 0.0001), blood HCO_3_^- ^22.3 ± 1.8 versus 22.6 ± 1.4 mmol/l (*P = *NS), BE -2.8 ± 2.1 versus -1.6 ± 1.2 (*P *< 0.01), serum phosphate 0.85 ± 0.2 versus 1.3 ± 0.5 mmol/l (*P *= 0.027), serum K^+ ^4 ± 0.2 versus 4.2 ± 0.3 mmol/l (*P = *NS) with KCl infusion 4 ± 0.2 versus 1.4 ± 1.5 mmol/hour (*P *< 0.0001). Protocol A required NaHCO_3 _and Na-phosphate infusion (8.9 ± 2.8 mmol/hour and 5 g/day, respectively) while protocol B allowed one to stop both supplementations. Systemic and circuit Ca^2+ ^were easily maintained in the target range with both protocols.

## Conclusion

Although needing confirmation in an adequate number of patients, protocol B was able to provide a buffer balance more positive than protocol A and allowed one to adequately control the A-B status without additional NaHCO_3 _infusion and in the absence of alkalosis, despite the use of a standard HCO_3_^- ^concentration HF solution. Furthermore, the combination of a phosphate-containing replacement fluid appeared effective to prevent hypophosphatemia. Finally, the use of a mathematical model allowed predicting the effects of different replacement solutions and/or RCA-CVVH settings on the mass balance of the main solutes.

